# Early complications after laparoscopic resection of choledochal cyst

**DOI:** 10.1007/s00383-019-04489-y

**Published:** 2019-05-27

**Authors:** Bing Zhang, Dianming Wu, Yifan Fang, Jianxi Bai, Wenhua Huang, Mingkun Liu, Jiancai Chen, Le Li

**Affiliations:** 1 Department of Pediatric Surgery, Fujian Provincial Maternity and Children’s Hospital, Fuzhou, 350001 China; 2 Department of Pediatric Surgery, Guangzhou Women and Children’s Medical Centre, Guangzhou, 510623 China

**Keywords:** Choledochal cyst, Laparoscopy, Complications

## Abstract

**Purpose:**

To investigate the causes and treatments of early complications involving laparoscopic radical resection of choledochal cyst and summarize the experience.

**Methods:**

Children with choledochal cyst treated by laparoscopy in the Department of Pediatric Surgery, Fujian Provincial Maternity and Children’s Hospital, and Guangzhou Women and Children’s Medical Centre, from March 2016 to May 2018, were retrospectively analysed. Demographics, causes and treatments of early complications, liver function analysis and ultrasonography were collected.

**Results:**

In total, 231 cases were included; 204 were Type I (156 Type Ia and 46 Type Ic) and 27 were Type IV. No mortality was observed, and 224 cases were successfully laparoscopically operated, while 7 cases were converted to open surgery. Fifteen cases of postoperative developed biliary fistula. There were jejunal Roux loop obstruction in 2 cases and multiple intussusception, anastomotic stenosis after hepaticojejunostomy, residual of choledochal cyst and pancreatic fistula in one each. Patients were followed up ranging from 4 months to 48 months (12.6 ± 0.3 months on average). Postoperative ALT, AST, GGT, TBIL and DBIL all returned to normal during this time. Ultrasonography indicated 5 cases of widened Glisson’s sheath and 1 case of intrahepatic hyperdense shadow.

**Conclusion:**

Early complications of laparoscopic radical resection of choledochal cyst can be minimized by properly managing preoperative indications and contraindications, carefully interpreting the magnetic resonance cholangiopancreatography results and accumulating experience by the surgeons.

## Introduction

Choledochal cyst frequently occurs in Asian, mainly in girls, and 80% of patients have their onset in childhood. Infants and younger children often demonstrate painless jaundice, while older children have recurrent abdominal pain [1]. It is a common disease in departments of paediatric surgery. With the development of imaging techniques and the prenatal screening, an increasing number of asymptomatic cases have been diagnosed [2]. Unless completely resected, the cyst can lead to recurrent symptoms and malignant transformation. Therefore, radical resection of choledochal cysts and Roux-en-Y hepaticojejunostomy are the ultimate effective treatments [3]. Laparoscopic resection of choledochal cyst has been widely used, but its early complications occurred frequently and should be given more attention. The purpose of this study is to analyse the causes of early complications after laparoscopic resection of choledochal cyst and summarize the experience of treatments.

## Materials and methods

This study presents a retrospective review of paediatric patients with choledochal cyst treated by laparoscopy in the Department of Pediatric Surgery, Fujian Provincial Maternity and Children’s Hospital, and Guangzhou Women and Children’s Medical Centre, from March 2016 to May 2018. Demographics such as gender, age, weight, clinical manifestation and Todani classification type [4] were collected. The surgery was performed with the four-hole method, as the trocars were located at the middle of the umbilicus, right hypochondrium, right side of the abdomen and left hypochondrium, respectively. After dissection of the gallbladder, bile was obtained for bacterial culture and amylase assay, and then, intraoperative cholangiography was performed. An monopolar electrocautery hook or a bipolar coagulation or ultrasonic scalpel was used to dissect the choledochal cyst. In cases of large cyst, the cyst was exposed by suspending the cystic wall using transabdominal sutures. The cyst was dissected down to the distal congenital bile duct dilatation to within the head of the pancreas, and it was then ligated. The upper part of the cyst was further dissected up to the level of the hilar duct and then removed at this level. The umbilical incision was extended to 2–2.5 cm, the jejunum that was 15–20 cm distal from the Treitz ligament was then extracted out of the incision and amputated, and a Roux-en-Y anastomosis was constructed, with a Roux loop length of approximately 15 to 30 cm. A retrocolic end-to-side hepaticojejunostomy was carried out laparoscopically. The mesenteric defects around the Roux loop in the transverse mesocolon and in the small bowel mesentery near the jejuno-jejunostomy routinely closed. A drainage tube was installed at the site of the right upper abdominal trocar. The operation time, blood loss and early complications were collected. Patients were followed up with liver function examinations and colour Doppler ultrasonography.

## Results

A total of 231 cases (42 boys and 189 girls) underwent laparoscopic choledochal cyst excision surgery during the study period. The age ranged from 1 to 139 month with an average of 7.2 ± 4.3 months; the weight ranged from 3.2 to 29.4 kg with an average of 7.3 ± 2.5 kg. Among them, 197 manifested abdominal pain, 135 had jaundice, 15 had acholic stools, 117 had abdominal masses, 132 had significantly elevated liver function indicators, and 79 had blood/urine amylase levels more than three times higher than normal. The Todani classification is as follows: 204 cases were Type I (156 Type Ia and 46 Type Ic) and 27 cases were Type IV [4].

No intraoperative and postoperative mortalities were observed, and 224 cases of patients were successively performed with laparoscopic operations; the operation time ranged from 2 to 8.3 h with an average of 3.8 ± 0.5 h and blood loss ranged from 20 to 240 ml with an average of 36 ± 10.2 ml; and intraoperative blood transfusion was needed in 13 cases. Seven cases was converted to open operation, of which 4 cases (one with biliary drainage after bile duct perforation, one with recurrent infections of choledochal cyst, one with a distal cyst residual after laparoscopic radical surgery and one with anastomotic stenosis after laparoscopic hepaticojejunostomy) had difficulties in identifying cystic walls or excessive bleeding when dissecting was due to severe local adhesions, oedema and thickening of the cystic walls. Two cases were due to the need for portoenterostomy, and the other one case was due to intraoperative damage to the hepatic artery.

Fifteen cases of postoperative biliary fistula were observed, of which 13 were cured by non-operative treatments and 2 underwent re-operation by open surgery. Between the 2 cases, one case was found to be caused by hepatic duct perforation and was cured after redo hepaticojejunostomy, and the other case was found accessory hepatic duct and was cured after anastomosis, the accessory hepatic duct to the jejunum. There were 2 cases of jejunal Roux loop stenosis and one case each for multiple intussusception, distal residue of choledochal cyst and anastomotic stenosis after hepaticojejunostomy. All of them underwent re-operation by open surgery. One case of pancreatic fistula was cured through conservative treatment (Table 1). The mean postoperative hospital stay ranged from 6 to 90 days with an average of 6.8 ± 2.3 days.

**Table 1:** Postoperative complications

Complication	Number	Clinical manifestation	Intraoperative findings	Treatment	Prognosis
Multiple intussusception	1	Vomiting yellow-green fluid Abdominal upright radiograph: intestinal obstruction	Multiple intussusceptions	Surgical reduction	Cured in 11 days
Biliary fistula	15	Stable body temperature; abdominal drainage gradually decreasing Biliary fluid is drained at 300–450 ml/day via abdominal drainage tube	None 1 case with hepatic common duct fistula; 1 case with the accessory hepatic duct	13 cases of drainage via abdominal drainage tube 1 case of re-operation with hepaticojejunostomy; 1 case of anastomosis between the accessory hepatic duct and the jejunum	Cured in 10–18 days Cured in 14–16 days
Anastomotic stenosis after hepaticojejunostomy	1	Manifesting paroxysmal abdominal pain 6 months after surgery, with occasional fever and jaundice; MRCP indicated intrahepatic bile duct dilatation; suspected of anastomotic stenosis after hepaticojejunostomy	Intraoperative cholangiography showed anastomotic obstruction	Redone hepaticojejunostomy after removing the original anastomotic stoma	Cured in 9 days
Jejunal Roux loop obstruction	2	Gastrointestinal decompression indicates non-biliary juice; biliary fluid is drained at 280–385 ml/day via abdominal drainage tube; excreting acholic stool; colour Doppler ultrasonography indicates Roux loop obstruction	The transverse mesocolon which bile branch passed through was too approached to the colon hepatic flexure, causing obstruction All the intestines of bile branch were moved to the above of the transverse colon, and the proximal end of the branch bile was compressed at the transverse mesocolon	Transverse mesocolon was dissected to move the Roux loop to the left of the non-stricture state Hepaticojejunostomy was re-performed	Cured in 10 days Cured in 17 days
Pancreatic fistula	1	Abdominal drainage tube discharges fluid at 310–1020 ml/day; abdominal drainage amylase level is at 1500–3430 U/l	None	Conservative treatment	Cured in 3 months
Residual cyst in the distal end of the common bile duct	1	Paroxysmal abdominal pain, fever and elevated blood amylase level 4 months after surgery; MRCP indicated residual cyst in the distal end of the common bile duct, with combination of stones	The residual cyst was about 3.5 × 3.0 cm	Resection of the residual cyst to within the head of the pancreas; removal of stones	Cured in 11 days

Mean follow-up time was 12.6 ± 0.3 months; liver function examination and colour Doppler ultrasonography were performed. Preoperative patients with normal alanine aminotransferase (ALT), aspartate aminotransferase (AST) and gamma-glutamyltransferase (GGT), who also performed normal during postoperative. Patients with elevated preoperative levels returned to normal after 3 weeks or 6 weeks or 12 weeks or 16 weeks or 20 weeks after surgery. Total bilirubin (TBIL) and direct bilirubin (DBIL) returned to normal 1–2 weeks after operation. Ultrasonography indicated 5 cases of widened Glisson’s sheath and 1 case of intrahepatic hyperdense shadow.

## Discussion

Choledochal cyst, also known as congenital biliary dilatation, includes partial dilatation of extrahepatic bile duct, common bile duct and pancreaticobiliary duct malformations and dilatation of the intrahepatic bile ducts, but it should strictly exclude acquired or secondary dilatations caused by bile duct stones or malignant tumours [5]. Choledochal cysts can lead to obstructive jaundice, liver damage, liver fibrosis and biliary bleeding, and the reflux of pancreatic juice into the biliary tract may cause a high incidence of biliary tract cancer; meanwhile, the entry of bile into the pancreatic duct may cause pancreatitis [6]. Laparoscopic radical surgery can reduce intraoperative bleeding and shorten fasting time and the length of hospitalization stay [7] and is thus currently a safe and effective method for the treatment of choledochal cyst [8, 9]. Due to its technical difficulties, complications occur inevitably. Analysis of the causes of complications and summarizing the experience of treatments can help to reduce complications and minimize the damage.

Biliary fistula is a common postoperative complication. Its cause is associated with anastomotic tension, anastomosis techniques and blood supply to anastomotic stomata. To prevent this, our experience has been that when dissecting the anterior wall of the cyst, it is recommended to avoid excessively dissecting tissues and completely remove the ulcerous lesions on the inner wall of the cyst. Most cases of biliary fistula can be cured by non-operative treatment. But if the abdominal drainage volume is large or there is no obvious decreasing trend under conservative treatment, it is recommended to re-operate approximately 3 days after initial surgery to avoid intra-abdominal tissue adhesion and oedema aggravation, which are not conducive to recovery after re-surgery. Those two patients with biliary fistula in our study, despite not manifesting fever or abdominal distension, whose drainaged bile were over 300 ml per day, underwent re-operation on the third day after initial surgery.

**Fig. 1:**
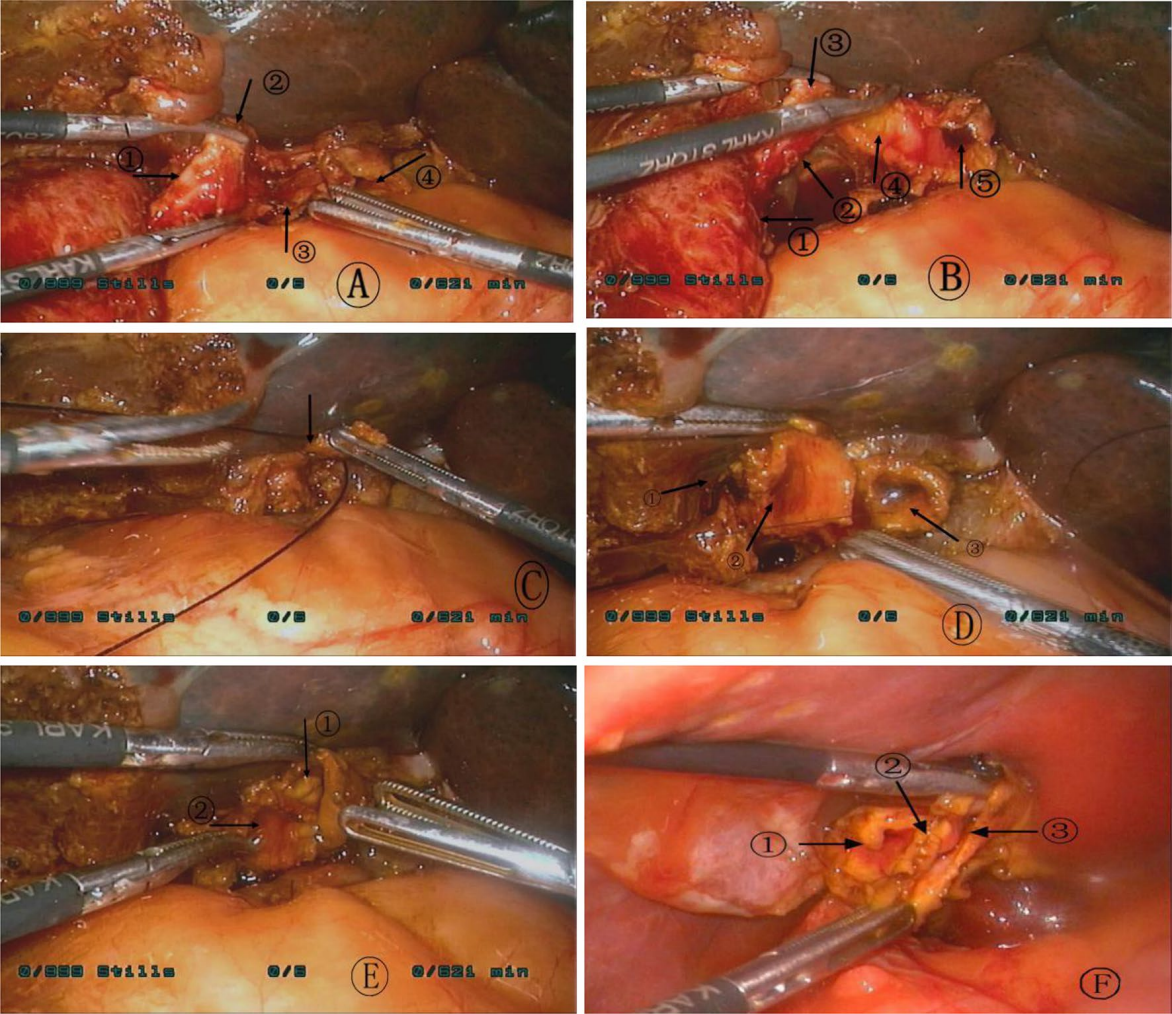
**a** Indicated by the arrow: ① distal end of the cystic duct; ② accessory hepatic duct; ③ proximal end of the cystic duct; ④ common hepatic duct. **b** Indicated by the arrow: ① gallbladder; ② distal end of the cystic duct; ③ accessory hepatic duct; ④ opened proximal end of the cystic duct; ⑤ common hepatic duct. **c** The diameter of the accessory hepatic duct is approximately 3 mm, making it difficult to anastomose, so the distal end of cystic duct is ligated at the location near the accessory hepatic duct (indicated by the arrow). **d** Indicated by the arrow: ① the ligated distal end of the cystic duct (near the accessory hepatic duct); ② the trimmed proximal end of the cystic duct; ③ common hepatic duct. **e** After the anastomosis between the accessory hepatic duct and the common hepatic duct (anastomosis between the trimmed cystic duct and common hepatic duct): ① common hepatic duct; ② the trimmed proximal end of cystic duct. **f** Another patient, after the anastomosis between the accessory hepatic duct and the common hepatic duct: ① accessory hepatic duct; ② suture surface; ③ common hepatic duct

The incidence of the accessory hepatic duct is 6% to 20%, and it is another important cause of biliary fistula. It is difficult to be detected by preoperative examination and is easy to be missed intraoperatively, which may result in bile duct infection, cholestasis and localized cirrhosis in the corresponding liver segment [10]. Therefore, we should be highly alert to the accessory hepatic duct when suspicious tubular structure occurs during dissecting the gallbladder or choledochal cyst and further clarification is needed to avoid complications. The accessory hepatic duct observed in this study was not found by preoperative examinations and was spotted as a tubular tissue approximately 2 to 3 mm in diameter connecting to the cystic duct during dissection of the cystic duct. The accessory hepatic duct was too small to be anastomosed with common hepatic duct. Therefore, it is recommended to ligature the cystic duct near the accessory hepatic duct, incise the residual cystic duct and anastomosis with the lateral wall of the common hepatic duct to form a new anastomosis, which is then anastomosed with the Roux loop (Fig. 1). In one case, a biliary fistula was found to be caused by the accessory hepatic duct. Due to the high discharge of bile drainage, it is not appropriate to perform ligation and instead, we anastomosed the accessory hepatic duct to the jejunum.

Bleeding is also a common complication. The causes mainly include trocar puncture injuries, injury of the gallbladder artery, hepatic artery, or portal vein, bleeding from cystic separation and abnormal coagulation. Our experience has been that in cases of large cysts or severe adhesion, the aspirator and the ultrasonic scalpel are used interchangeably, and coupled with suspending and pulling the cyst with sutures, the posterior wall of the cyst can be gradually dissected. Additional care should be taken on the portal vein and hepatic artery when dissecting the posterior wall of the cyst. It is reported [11] that puncturing the right hepatic artery when suturing the posterior wall during hepaticojejunostomy caused a massive haemorrhage on the third day after surgery, requiring surgical exploration. So it is recommended that when suturing the posterior wall, the anterior wall of the common hepatic duct should be suspended to expose the posterior wall so that vascular damage can be avoided. In cases of active postoperative bleeding after transfusion and correction of coagulopathy, re-operation should be conducted in time.

**Fig. 2:**
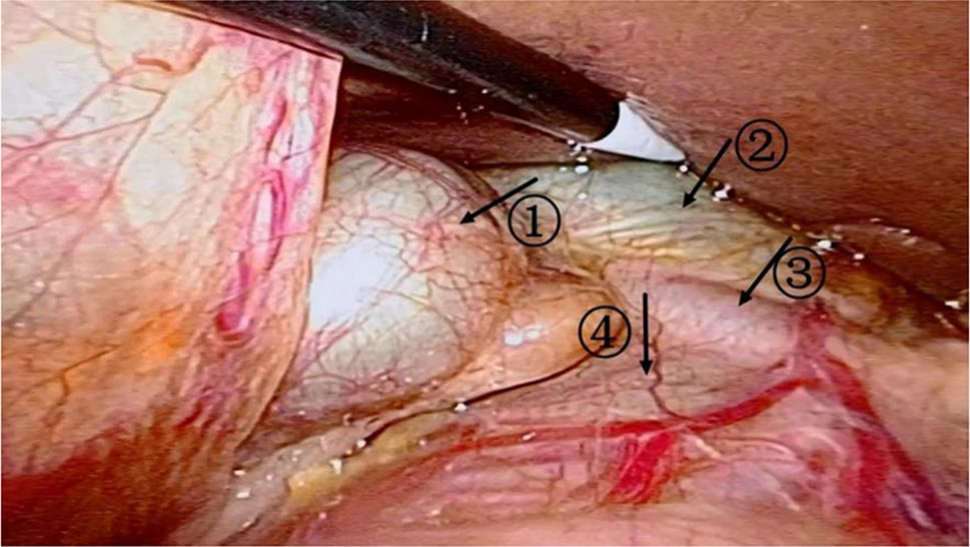
Indicated by the arrow: ① cystic duct; ② proximal end of the common hepatic duct; ③ ectopically presented right hepatic artery; ④ choledochal cyst

**Fig. 3:**
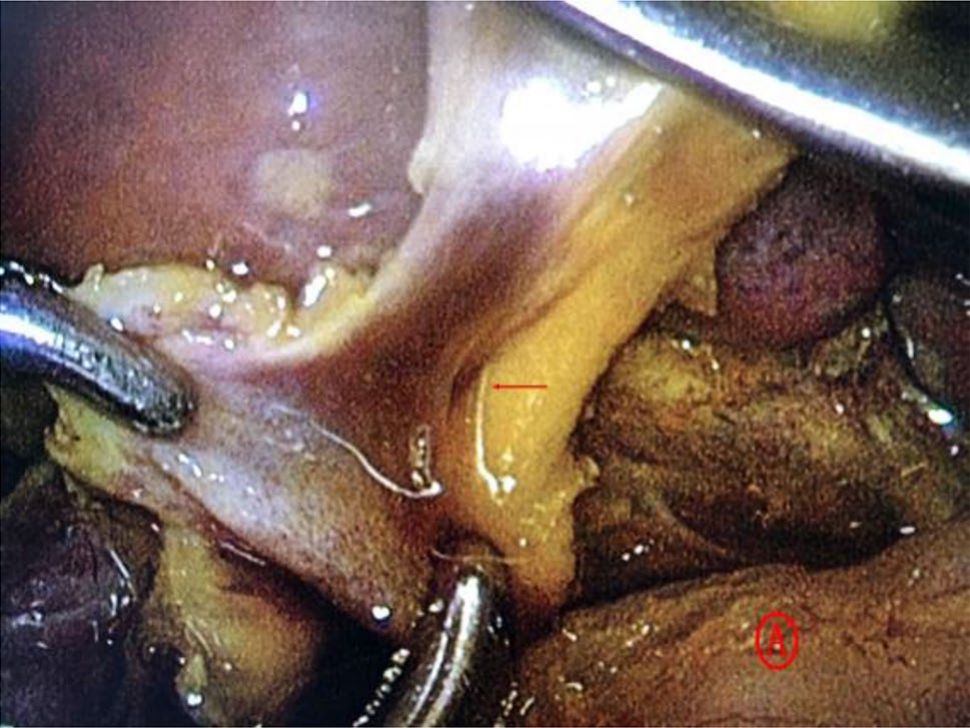
A. Indicated by the arrow is the fine opening of the common hepatic duct

**Fig. 4:**
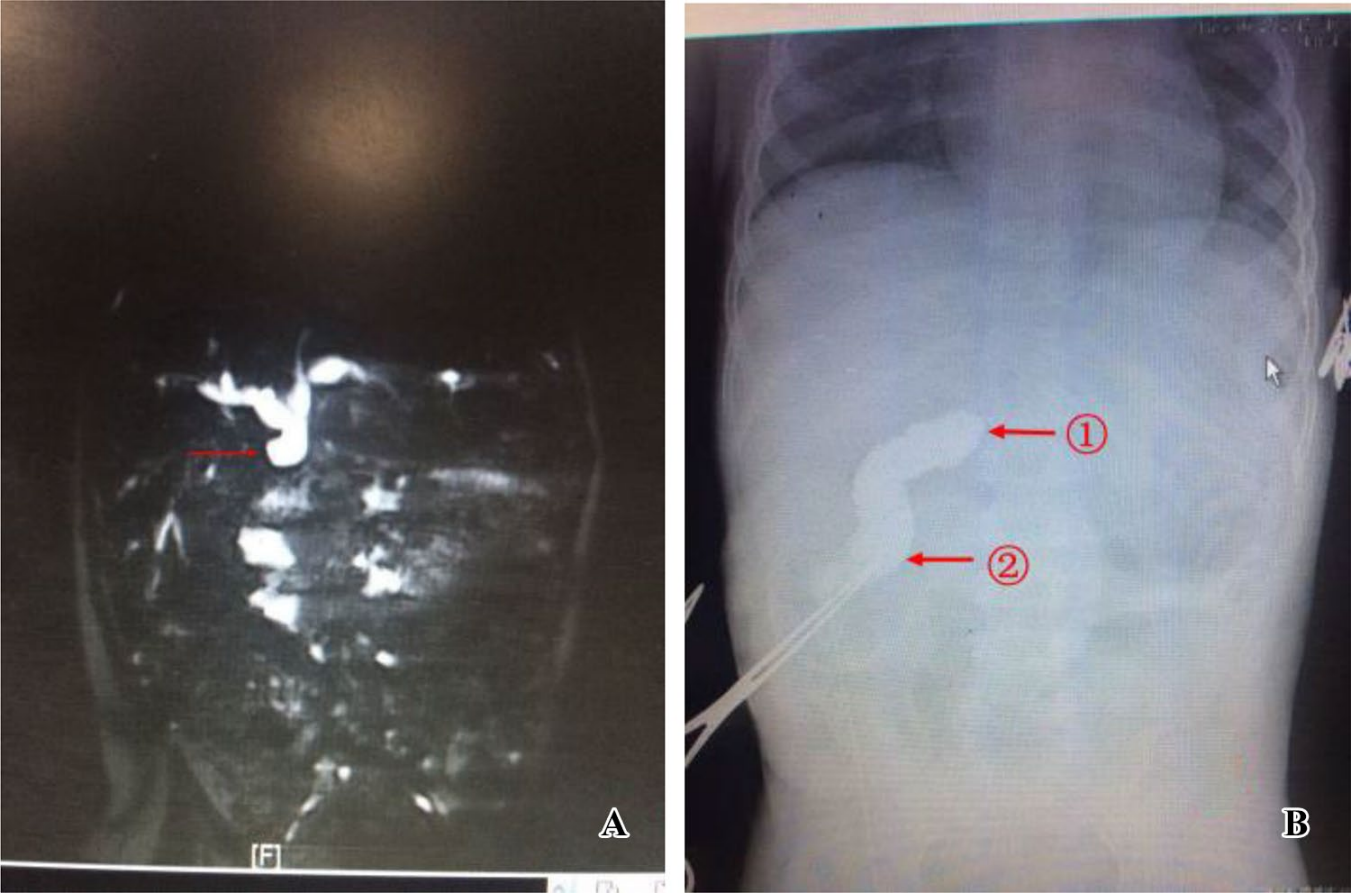
**a** MRCP examination; the arrows indicate anastomotic stenosis after hepaticojejunostomy, the significantly dilated proximal end of the common hepatic duct, and the left and right hepatic ducts. **b** Intraoperative contrast injection into the jejunal Roux loop, indicating: ① anastomotic stenosis after hepaticojejunostomy; ② the injection of contrast agent after the distal end of the duct of the Roux loop was blocked with an intestinal clamp

Biliary obstruction after bile duct reconstruction is not rare. The causes include aberrant right hepatic artery, combination with hepatic duct stenosis, anastomotic stenosis and jejunal Roux loop obstruction. It is recommended to re-operate as soon as possible to reduce liver damage. (1) Undetected right hepatic artery in front of the extrahepatic biliary tract is among the main causes of recurrent biliary obstruction after radical surgery. In biliary tract reconstruction, placing the hepatic artery behind the common hepatic duct (Fig. 2) to restore normal anatomy can avoid this complication [12, 13]. (2) Regarding common hepatic duct stenosis, analysis of preoperative imaging and carefully exploring stenosis of the common hepatic duct and the left and right hepatic ducts intraoperatively, the stenotic segment should be opened to normal level. Two patients aged approximately 2 months were found to have the common hepatic duct which was small to approximately 1 mm in diameter when dissecting the proximal end of the cyst. Although preoperative magnetic resonance cholangiopancreatography (MRCP) did not indicate proximal biliary stenosis or dilatation, intraoperative cholangiography showed that contrast did not enter the left or right hepatic ducts, so we suspected the concurrent common hepatic duct stenosis and opened the duct in the anterior wall. We found that the common hepatic duct was very thin and it is difficult to clearly identify the left and right hepatic ducts even under a magnified laparoscopic field of view; thus, we performed hepatic portal-jejunal anastomosis ultimately (Fig. 3). In this regard, our experience is that if the common hepatic duct is found small, even if the left and right hepatic ducts do not develop on intraoperative cholangiography, as long as the preoperative MRCP does not suggest proximal hepatic duct dilatation and the drainage volume of bile at the opening of common hepatic duct is found to be large during surgery, then we would not consider the combination of stenosis and would not recommend opening the anterior wall of the main hepatic duct. (3) The incidence of stenosis of choledochal-jejunal anastomosis after laparoscopic choledochal cyst excision was 1.6%. The main causes include small anastomosis, excessive tension, insufficient blood supply and inflammation or infection associated with biliary leakage [11, 14, 15]. In this study, anastomotic stenosis occurred in one case with biliary fistula 6 months after initial surgery, so the original anastomotic stoma was amputated and a new anastomosis was performed; the patient was eventually cured (Fig. 4). So we recommended that measures should be undertaken to ensure no tension on the stoma, good blood supply to the stoma, a sufficiently large stoma diameter (infant ≧ 1 cm, and newborn ≥ 0.5 cm) and that the technique of anastomosis should be improved to avoid stenosis. (4) The incidence of jejunal Roux loop obstruction is not high, mostly related to the twisting caused by excessively long Roux loops [16]. However, the two cases observed in this study occurred in the region at which the Roux loop crosses the transverse mesocolon. They manifested paroxysmal abdominal pain on the first day after radical surgery, and abdominal drainage tube drained biliary fluid. After non-operative treatment, the abdominal pain was not relieved, ultrasonography indicated jejunal Roux loop dilation, and the patients were re-operated on the third day after surgery. Our experience is that it is necessary to determine the location of the common hepatic duct, so the transverse mesocolon region is identified to ensure the shortest and smoothest path of the Roux loop. Then, the dissection is performed in the avascular region of the transverse mesocolon region with an ultrasonic scalpel, and after passing across the transverse mesocolon region, the Roux loop was lifted again to ensure that no twisting or angulation was present. The seromuscular layer of the mesocolon passing across the transverse mesocolon region and the transverse mesocolon were fixed with sutures. After radical surgery, if the patient manifests paroxysmal abdominal pain, a large volume of bile drainage through the abdominal drainage tube, no biliary outflow from gastrointestinal decompression, and the excretion of acholic stools, we should be highly alert to the Roux loop obstruction.

Pancreatic fistula occurs mainly because the distal end of the common bile duct cyst is not close enough to the common wall of the cyst wall. The diagnostic criterion of the International Pancreatic Research Group for pancreatic fistula is that > 3 days after surgery, the amylase concentration in any amount of drainage fluid is more than three times higher than the upper limit of a normal serum amylase concentration, which is related to the prognosis. An abdominal fluid amylase level of over 2000 U/l on the first postoperative day may help to diagnose pancreatic fistula [17, 18]. If pancreatic fistula is suspected after surgery, an additional fine plastic tube can be inserted through the abdominal drainage tube, through which normal saline is continuously perfused and then drained via a thick drainage tube. If the flushing is effective, conservative treatment can be considered; if after the flushing, the abdominal symptoms are aggravated, the body temperature continues to rise. In such a case, flushing through ultrasound-guided intubation or re-operation should be performed.

**Fig. 5:**
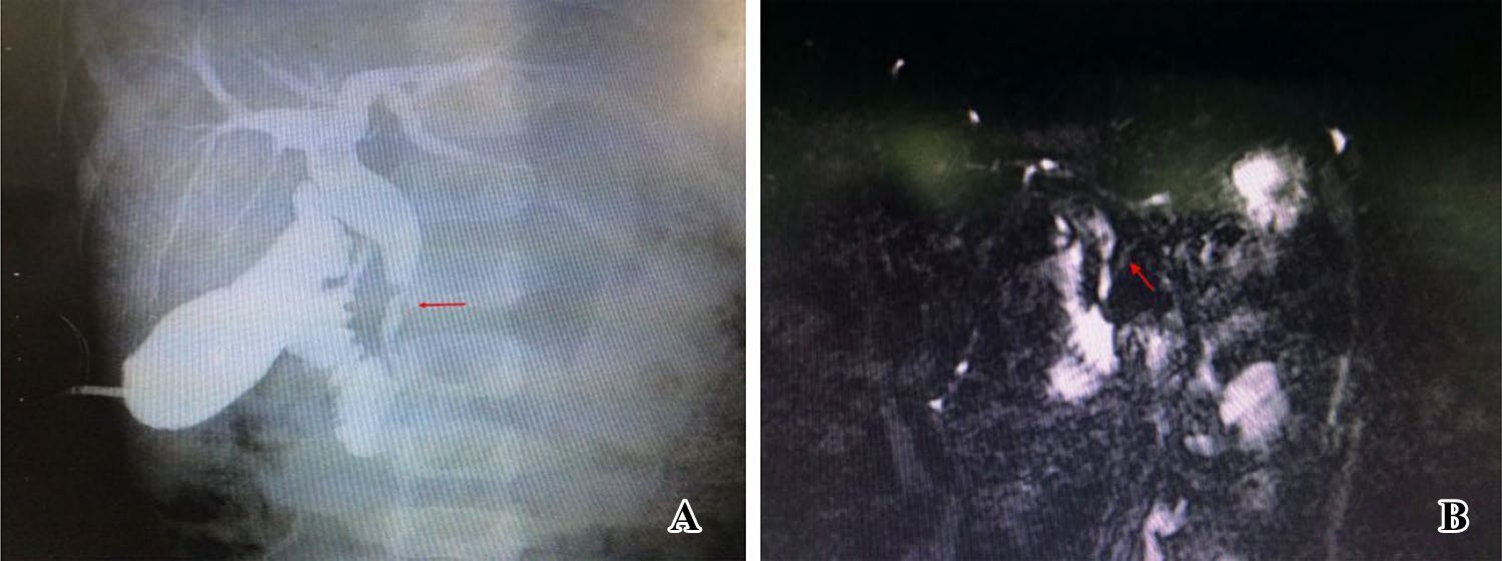
**a** Intraoperative cholecystography; the arrow indicates the pancreatic duct. **b** Preoperative MRCP examination; the arrow indicates the pancreatic duct

Postoperative pancreatitis is rare and associated with abnormal pancreatic ducts, such as residual cyst in pancreas [19]. At the early stage in this study, there was a case in which the distal end of the cyst was not completely dissipated by laparoscopic operation to avoid the injury of pancreatobiliary common duct and thus would be in the need for re-operation. In the later cases, the MRCP was read carefully before surgery, and the intraoperative cholangiography was used to identify the pancreatic duct. Pancreatic duct injury could be avoided when dissecting the cyst to the distal. And then suturing after full washing, no similar complications appear (Fig. 5).

Although this study is a two-centre, large-sample study, there are still some limitations: the sample is a continuous sample and there is selective bias. Follow-up time is too short; long-term complications such as anastomotic stricture, biliary calculi, reflux cholangitis and so on need to be further followed up.

## Conclusion

In summary, laparoscopic radical resection of the choledochal cyst has become a mature technique; despite high surgical difficulty and various complications, the proper preoperative management of indications and contraindications and careful interpretation of the MRCP results with the surgeon’s accumulation of surgical experience can minimize surgical complications.
